# Ex Vivo Lung Perfusion in the Rat: Detailed Procedure and Videos

**DOI:** 10.1371/journal.pone.0167898

**Published:** 2016-12-09

**Authors:** Giulia Alessandra Bassani, Caterina Lonati, Daniela Brambilla, Francesca Rapido, Franco Valenza, Stefano Gatti

**Affiliations:** 1 Center for Surgical Research, Fondazione IRCCS Ca’ Granda—Ospedale Maggiore Policlinico, Milan, Italy; 2 Center for Preclinical Investigation, Dipartimento di Anestesia, Rianimazione ed Emergenza Urgenza, Fondazione IRCCS Ca’ Granda—Ospedale Maggiore Policlinico, Milan, Italy; 3 Department of Pathophysiology and Transplantation, University of Milan, Milan, Italy; Cedars-Sinai Medical Center, UNITED STATES

## Abstract

Ex vivo lung perfusion (EVLP) is a promising procedure for evaluation, reconditioning, and treatment of marginal lungs before transplantation. Small animal models can contribute to improve clinical development of this technique and represent a substantial platform for bio-molecular investigations. However, to accomplish this purpose, EVLP models must sustain a prolonged reperfusion without pharmacological interventions. Currently available protocols only partly satisfy this need. The aim of the present research was accomplishment and optimization of a reproducible model for a protracted rat EVLP in the absence of anti-inflammatory treatment. A 180 min, uninjured and untreated perfusion was achieved through a stepwise implementation of the protocol. Flow rate, temperature, and tidal volume were gradually increased during the initial reperfusion phase to reduce hemodynamic and oxidative stress. Low flow rate combined with open atrium and protective ventilation strategy were applied to prevent lung damage. The videos enclosed show management of the most critical technical steps. The stability and reproducibility of the present procedure were confirmed by lung function evaluation and edema assessment. The meticulous description of the protocol provided in this paper can enable other laboratories to reproduce it effortlessly, supporting research in the EVLP field.

## Introduction

Lung transplantation is the only therapeutic option for patients with end-stage organ failure. However, graft shortage is a major limiting factor to clinical success. Indeed, it is estimated that only 15–20% of potential lungs from multiorgan donors are currently suitable for transplantation [1].

Ex vivo lung perfusion (EVLP) is a promising strategy to cope with this problem. This technique was initially developed by Steen and co-workers to evaluate lungs from donation after cardiac death [2]. Subsequently, EVLP was implemented as a method to preserve and repair marginal organs prior to transplant [3–5].

Many animal models of EVLP have been developed to support clinical use. Because of their appropriate human-like lung size, large animals, such as pigs, have been broadly used to exploit this system [6, 7]. While these models were crucial to improve specific technical skills, small animal models can offer better means to identify the pathophysiological bio-molecular changes associated with ex vivo perfusion [8]. This kind of information is needed as the mechanism(s) underlying beneficial effects of extracorporeal reconditioning on donor lungs are largely unknown. Further, it is equally important to identify potential damage of EVLP. Analysis of molecular changes that occur in the lung at definite procedure steps can help correction through targeted interventions.

The present investigation is part of a broader research project aimed at identification and characterization of molecular changes induced in the lung by ex vivo perfusion and, eventually, evaluation of different therapeutic interventions. Set up and optimization of a simple and reproducible model for a normothermic and protracted rat EVLP was the main focus in this study. Indeed, a rat EVLP protocol that is stable over time is a solid foundation to any further research in the field. Particular efforts were devoted to safely prolong perfusion time without using pharmacological treatments. To accomplish this purpose, several technical issues associated with lung damage were identified and implemented. With the reliable support of videos, this study provides a detailed identification of the critical steps connected with the procedure and their successful management. The meticulous description of our protocol will enable other laboratories to reproduce it effortlessly, reducing the number of experiments in full respect of the 3Rs principles [9].

## Materials and Methods

### Animals

The experiments were performed in strict accordance with the recommendations in the Guide for the Care and Use of Laboratory Animals of the National Institutes of Health, at the Center for Preclinical Investigation, Fondazione IRCCS Ca’ Granda Ospedale Maggiore Policlinico, Milan, Italy. The experimental protocol was approved by the Italian Institute of Health (Permit Number: 5/13).

Adult Sprague–Dawley male rats (Charles River, Calco, Lecco, Italy) weighing 270–330 g were housed in a ventilated cage system (Tecniplast S.p.A., Varese, Italy) at 22 ± 1°C, 55 ± 5% humidity, on a 12 h dark/light cycle, and were allowed free access to rat chow feed and water ad libitum.

### Reagents and instruments

The reagents and instruments used in this protocol are shown in Tables 1 and 2.

**Table 1:** Reagents employed.

Reagent	Manufacturer
Thiopental sodium, 0.5 g	Inresa Arzneimittel GmbH, Freiburg, Germany
Heparin, 5000 UI/ml	Pharmatex Italia S.r.l., Milano, Italy
Perfadex®	XVIVO Perfusion AB, Gotebörg, Sweden
NaHCO_3_, 8.4%	S.A.L.F. S.p.A. Laboratorio Farmacologico, Bergamo, Italy
CaCl_2_, 1.36 mEq/ml	Bioindustria L.I.M. S.p.A., Novi Ligure, Italy
Mucasol™ universal detergent	Sigma-Aldrich, St. Louis, Missouri, United States
Basic Glutaster	Farmec, Settimo di Pescantina, Italy
Albumin, 0.2 g/ml 20% immuno	Baxter S.p.A., Roma, Italy
NaCl, 0.9%	Baxter S.p.A.
Amphotericin B, 250 μg/ml	Life Technologies, Foster City, California, United States
Glucose, 33%	B. Braun, Melsungen, Germany
KCl, 2 mEq/ml	B. Braun
K_3_PO_4_, 2 mEq/ml	S.A.L.F. S.p.A. Laboratorio Farmacologico
MgSO_4_, 0.8 mEq/ml	S.A.L.F. S.p.A. Laboratorio Farmacologico
Cefazolin, 0.1 g/ml	Pfizer Italia S.r.l., Latina, Italy
Gas mixture of CO_2_ (5%), O_2_ (21%) and N_2_ (74%)	Sapio S.r.l., Monza, Italy
LB agar	Sigma-Aldrich

**Table 2:** Instruments employed.

Instrument	Manufacturer
Temperature control unit HB 101/2 RS	Panlab Harvard Apparatus, Barcelona, Spain
Surgical heating pad	Panlab Harvard Apparatus
Isolated lung perfusion systems size 2	Hugo Sachs Elektronik, Harvard Apparatus GmbH, March-Hugstetten, Germany
Roller pump	Ismatec, Wertheim, Germany
Heat exchanger Ecoline E 103	Lauda Dr. R. Wobser Gmbh & Co. Kg, Lauda-Konigshofen, Germany
Harvard model 683 small animal ventilator	Harvard Apparatus, Holliston, Massachusetts, United States
Automatic blood gas analyzer ABL 800 FLEX	A. De Mori Strumenti, Milano, Italy
Data acquisition software Colligo	Elekton, Milano, Italy
Analytical balance ABT 100-5M	KERN & SOHN GmbH, Balingen, Germany
Oven	LTE Scientific, Greenfield, United Kingdom
14 G tube	Delta Med S.p.A, Viadana, Italy
2.0/2.5 mm pulmonary cannula	Hugo Sachs Elektronik
Double-headed surgical microscope OPMI 1-F	Zeiss West Germany, Oberkochen, Germany
1.3 mm temperature probe	Panlab Harvard Apparatus
5F Swan-Ganz catheter	Pulsion Medical System SE, Feldkirchen, Germany
Laminar flow hood	Gelaire ICN Biomedicals, Sydney, Australia
Filtropur V 100 0.22 μm	Sarstedt, Numbrecht, Germany

### Anesthesia and pre-operative preparation

All procedures were performed under sterile conditions.

Rats were anesthetized with an intraperitoneal injection of 80 mg/kg thiopental sodium. The animals, placed on a surgical heating pad in supine position, received an intravenous injection of 600 IU heparin. Rectal temperature was continuously monitored.

### Isolated lung perfusion system

The perfusion circuit consisted of a glass chamber, a roller pump, a reservoir, a bubble trap, and silicon tubing. The glass chamber and reservoir were equipped with a water-jacket to control the perfusate temperature through a heat exchanger. A small animal ventilator was used.

### Gas analysis of perfusate samples

Inflow and outflow perfusate composition, from reservoir and left auricle respectively, was hourly monitored with an automatic gas analyzer.

### Mean pulmonary artery and airway pressure measurements

During the whole experiment, mean pulmonary artery pressure (PAP), positive end expiratory pressure (PEEP), and peak inspiratory pressure (Ppeak) were continuously recorded via an amplifier connected to the pulmonary and tracheal cannulae.

Total pulmonary vascular resistance (TPVR) was calculated using the formula: $\frac{80\times (PAP-WP)}{PAF}$, where PAF is the pulmonary artery flow and WP the wedge pressure (set to 0).

### Wet-to-dry weight ratio

At the end of the ex vivo perfusion, the right superior lobe was weighed with an analytical balance and dried in an oven at 50°C for 24 h. Wet-to-dry ratio (W/D) was calculated and used as an index of pulmonary edema. Lungs harvested from rats in basal conditions (n = 10) were used as controls.

### Statistical analysis

All results are presented as mean ± standard error of the mean (SEM) or as the median [first quartile–third quartile]. Statistical analysis was performed using t-test or one-way analysis of variance (ANOVA) for repeated measures, followed by Tukey’s multiple comparison test to evaluate differences at each time points. Non-linear regression analysis was used to investigate correlation, whereas agreement between measurements performed on samples collected from left auricle and reservoir was explored using linear regression and Bland-Altman analysis. Limits of agreement were computed as the average of the differences (bias) plus and minus 2 times the standard deviation. A probability value <0.05 was considered significant. Data were analyzed using Sigma Stat 11.0 dedicated software (Systat Software Inc., San Jose, United States).

## Results

Over a 5 month-period, 44 rats were subjected to heart-lung block harvest. Two animals were excluded, one because of air embolism during cannulation of the pulmonary artery and the other because of lung damage during harvest. The ex vivo procedure was performed on 42 lungs; 5 of them were discarded, 4 because of air embolism during EVLP and one due to a procedural mistake. Thirty-seven lungs were successfully perfused for 180 min and were included in a randomized single-blind study; 27 of these organs were subjected to different therapeutic interventions as part of a larger research program. Surgical outcomes and functional parameters from the 10 untreated experiments are the subject of the present research and are described in detail in this article.

### Surgical procedure

#### In situ lung function evaluation

The whole procedure was conducted with the aid of a surgical microscope. The trachea was cannulated with a 14 G tube and monitoring of airway pressure was started. The abdomen was entered with a midline xiphopubic incision and inferior vena cava was sectioned. The surgical heating pad was then removed and rats were left at room temperature (RT). Subsequently, continuous positive airway pressure (CPAP) with PEEP of 3 cmH_2_O and 100% oxygen 0.3 L/min was started. The thorax was entered through a midline incision and a recruitment maneuver (RM) aimed at expansion of pulmonary atelectasis was performed using 25 ml/kg air at RT. One ml-aliquots of ambient air were insufflated into the lungs to measure total lung capacity (TLC), defined as the volume at which the pressure-volume curve shows an upper inflection point (overstretch) [10]. A volume corresponding to half TLC was used to calculate the basal elastance (S1 Video).

#### In situ lung perfusion

Diaphragm, pulmonary ligaments and pericardium were sectioned and thymus removed. Ascending aorta and pulmonary artery were encircled. Pulmonary artery cannulation was performed with a 2.0/2.5 mm cannula through a right ventricular incision, after careful removal of air bubbles. Atrial auricles and heart apex were resected to vent pulmonary circulation (S2 Video).

CPAP was replaced by volume controlled ventilation (VCV) using ambient air, with tidal volume (VT) of 6 ml/kg, PEEP of 2 cmH_2_O and respiratory rate (RR) of 10 bpm. Lungs were then flushed at 25 cm H_2_O pressure with 60 ml/kg of ice-cold Perfadex® buffered with 10 mEq/L of 8.4% NaHCO_3_ and 0.8 mEq/L of CaCl_2_. At the end of flushing, ventilation was stopped and lungs were kept inflated during retrieval (S3 Video).

#### Heart-lung block harvest

Diaphragm and suprahepatic vena cava were dissected and lung ligaments sectioned. The lungs were gently turned upside down with two cotton swabs to perform posterior dissection. Trachea was isolated from esophagus and cervical vasculature. Harvest of the heart-lung block was performed maintaining both tracheal and pulmonary cannulae in place. After harvest, the lungs were still inflated and appeared homogeneously perfused (S4 Video). At this stage, we recommend particular caution in handling lungs, avoiding torsion of the hilar structure.

#### Surgical outcomes

**Fig 1:**
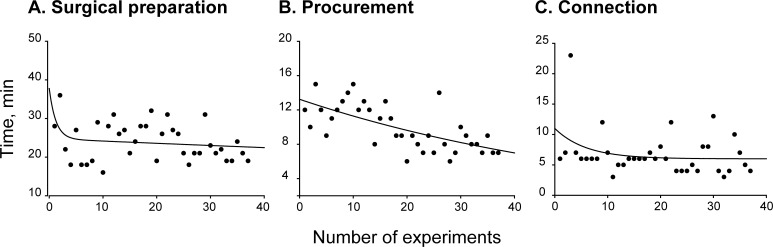
Learning curves. (A) Surgical preparation time (from heparin administration to flushing; R = 0.291, p = 0.397). (B) Procurement time (from flushing to harvest; R = 0.660, p = 0.0003); (C) Connection time (from harvest to lung connection to the circuit; R = 0.299, p = 0.370). Nonlinear regression analysis.

Time periods required for surgical preparation (from heparin injection to flushing), heart-lung block procurement (from flushing to harvest), and lung connection (from harvest to connection to the circuit) were examined as indexes of technical improvement. Nonlinear regression analysis of 37 experiments over a 5 month-period showed a significant decrease in procurement time (R = 0.660, p = 0.0003, Fig 1A), whereas duration of surgical preparation and connection steps remained unchanged (R = 0.291, p = 0.397, Fig 1B and R = 0.299, p = 0.370, Fig 1C, respectively). The 10 rats (300 ± 3 g) included in the present paper were subjected to 36.6 ± 1.9 min total operation time (from heparin injection to lung harvest). Specifically, time for surgical preparation was 26.9 ± 1.4 min, flushing 1.3 ± 0.1 min, procurement 9.8 ± 0.8 min, cold ischemia (from flushing to ex vivo perfusion) 15.9 ± 1.0 min, and connection 7.3 ± 1.1 min. Start core temperature, recorded during systemic heparinization, was 35.6 ± 0.3°C and basal compliance was 0.38 ± 0.01 ml/cmH_2_O.

### EVLP procedure

#### Perfusion solution

The present protocol used an acellular perfusion fluid with extra-cellular electrolyte composition. The solution was freshly prepared in sterile conditions in a laminar flow hood, using: albumin, NaCl, Perfadex^®^, amphotericin B, glucose, CaCl_2_, KCl, K_3_PO_4_, MgSO_4_, and heparin. Although two separate lots of albumin with different sodium concentrations were used, electrolyte composition was maintained stable. Characteristics of the two solutions were: osmolality 294 and 286 mOsm/kg; albumin 8.8 and 8.9 g/dl; total protein 9.4 g/dl for both lots. The perfusion fluid was filtered using a vacuum filtration unit to remove any particulate impurity or bacterial contamination and stored for a maximum of 5 days to avoid bacterial growth.

Before each experiment, the fluid was added with cefazolin and NaHCO_3_, whereas no treatment with corticosteroids or other anti-inflammatory drugs was performed.

#### Isolated lung perfusion system

The heart-lung block was placed onto the glass chamber modified to let the lung dorsal side lay on a modeled ad hoc, perforated surface. The lungs were connected to the circuit through the pulmonary cannula and to the ventilator through the tracheal cannula. The chamber was closed with a polystyrene lid to maintain humidity. Temperature inside the chamber was recorded with a 1.3 mm probe. In preliminary experiments, perfusate temperature was measured using a 5F Swan-Ganz catheter probe, positioned in the pulmonary cannula. The observation was that, in order to obtain a lung temperature of 37.5°C, the heat-exchanger had to be set at 42°C. Indeed, we found that the temperature recorded in the pulmonary cannula was considerably lower relative to that set in the instrument (data not shown). This information is very important for an appropriate heat-exchanger setting.

The circuit was primed with 110–140 ml of perfusion fluid according to rat weight. In our experience, correct priming of the perfusion system is essential to avoid air embolism. The EVLP system is showed in S5 Video. At the end of each experiment, the perfusion apparatus was washed with H_2_O_2_, Mucasol^TM^ universal detergent and Basic Glutaster.

#### Isolated lung perfusion protocol

**Fig 2:**
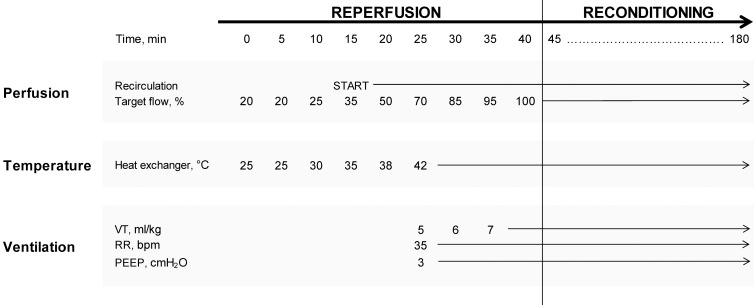
Protocol timeline. Schematic overview of EVLP protocol. Abbreviations: VT, tidal volume; RR, respiratory rate; PEEP, positive end expiratory pressure.

Lung perfusion was started when pH of the solution reached 7.2. As shown in Fig 2, ex vivo perfusion was maintained for 180 min and included 2 phases. The first 40-min phase, named “reperfusion” (also denoted “stabilization” or “ramp-up”), included a gradual rise of PAF, temperature, and VT. Initial PAF was set at 20% of 6 ml/min/g of predicted lung weight (PLW), calculated using the formula: *PLW* (*g*) = 0.0053 × *body weight* (*g*) − 0.48 [11]. PAF was progressively increased to 100%, equivalent to 5.71 and 7.61 ml/min for rats weighing 270 and 330 g, respectively. Temperature was gradually increased from 25° to 42°C in 25 min. When the lung reached a normothermic state, VCV ventilation was started using a gas mixture of CO_2_ (5%), O_2_ (21%) and N_2_ (74%). RR was set at 35 bpm, PEEP at 3 cmH_2_O, and initial VT at 5 ml/kg. Over the next 10 min, VT was increased to 7 ml/kg (S6 Video). During the first 15 min of the reperfusion phase, the perfusate draining from the left atrium was collected and discarded every 5 min. Thereafter, the outflow perfusate was let to re-circulate into the reservoir.

The second phase, named “reconditioning” (also denoted “steady-state”), began when all parameters reached their target value. Two RMs were applied at 40 and 175 min by manually inflating the lungs at 20 cmH_2_O with ambient air (S6 Video). During this procedure, PAF was reduced to half the set flow rate.

At the end of ex vivo perfusion, biopsies were performed for edema evaluation and 1 ml perfusate was dispensed to LB agar plates. Absence of bacterial growth after 4 days at 37°C indicated no contamination.

### EVLP effects

#### Gas analysis of perfusate samples

Characteristics of the perfusion solution at priming (time 0) and in samples collected from the auricle at 60, 120, and 180 min are shown in Table 3. All measured parameters changed over time (p<0.001). Of note, glucose concentration decreased from 192 ± 3 mg/dl at priming to 175 ± 2 mg/dl at 180 min, whereas lactate increased from 0.3 ± 0.0 mmol/L at 60 min to 0.9 ± 0.1 mmol/L at 180. Linear regression analysis showed a significant correlation for all the measured parameters in samples withdrawn from the auricle and those from the reservoir (R≥0.801; p<0.001), with the only exception of pO_2_ (R = 0.0937, p = 0.622) (S1 Fig, panel A). Bland-Altman plots showed that electrolyte and metabolite concentration fell within the limits of agreement (S1 Fig, panel B), whereas a constant error in pO_2_ and pH measurements was revealed.

**Table 3:** Changes over time in perfusion fluid parameters.

	Perfusion time, min			
	0	60	120	180
**pH**	7.224 ± 0.006	7.340 ± 0.007^a^	7.336 ± 0.006^a^	7.331 ± 0.008^a^
**BE,** mmol/L	-11.8 ± 0.4	-11.4 ± 0.4	-12.1 ± 0.3^b^	-12.7 ± 0.4^a^^,^^b^
**pO**_**2**_**,** mmHg	175 ± 2	155 ± 3	154 ± 3^a^	153 ± 3^a^
**pCO**_**2**_**,** mmHg	36.2 ± 0.8	25.3 ± 0.5	24.2 ± 0.5^a^	23.6 ± 0.5^a^^,^^b^
**K**^**+**^**,** mmol/L	4.6 [4.5–4.8]	4.7 [4.7–4.9]^a^	4.7 [4.7–4.9]^a^	4.8 [4.7–4.9]^a^
**Na**^**+**^**,** mmol/L	145 ± 0	146 ± 0^a^	147 ± 0^a^^,^^b^	148 ± 0^a^^,^^b^^,^^c^
**Ca**^**2+**^**,** mmol/L	0.67 ± 0.01	0.61 ± 0.01^a^	0.61 ± 0.01^a^	0.61 ± 0.01^a^
**Cl**^**-**^**,** mmol/L	107 ± 2	110 ± 1^a^	112 ± 1^a^^,^^b^	113 ± 1^a^^,^^b^
**Glc,** mg/dl	192 ± 3	186 ± 2	181 ± 2^a^	175 ± 2^a^^,^^b^
**Lac,** mmol/L	0.0 ± 0.0	0.3 ± 0.0^a^	0.6 ± 0.1^a^^,^^b^	0.9 ± 0.1^a^^,^^b^^,^^c^

Perfusate composition was monitored hourly using an automatic gas analyzer. Results are expressed as mean ± SEM or as the median [first quartile–third quartile]. One-way repeated measures ANOVA; p value was <0.001 for each parameter considered. Tukey’s multiple comparison test

^a^ p<0.05 vs time 0

^b^ p<0.05 vs time 60

^c^ p<0.05 vs time 120. Abbreviations: BE, base excess; pO_2_, partial pressure of oxygen; pCO_2,_ partial pressure of carbon dioxide; Glc, glycemia; Lac, lactate concentration.

#### Mean pulmonary artery and airway pressures

**Fig 3:**
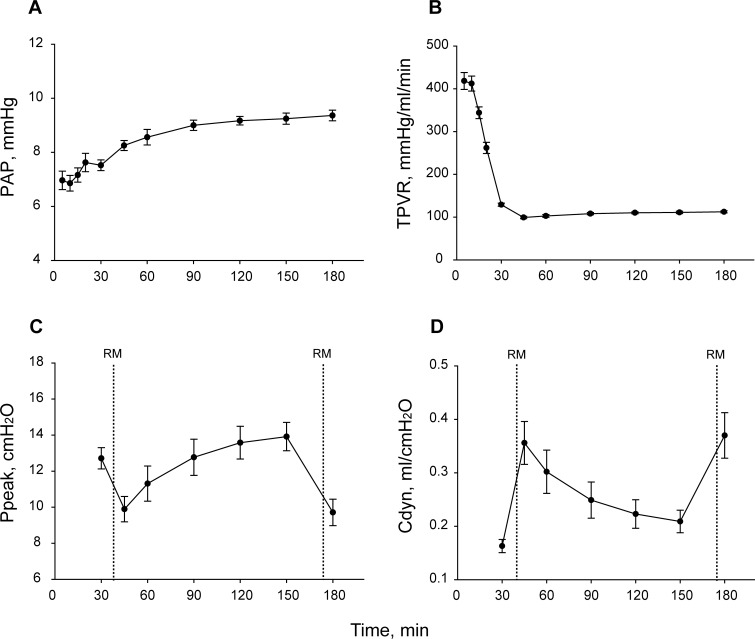
Lung parameter changes during EVLP. (A) Pulmonary artery pressure (PAP). (B) Total pulmonary vascular resistance (TPVR). (C) Peak inspiratory pressure (Ppeak). (D) Dynamic compliance (Cdyn). Pressure recruitment maneuvers (RMs) were performed at 40 and 175 min. Results are expressed as the mean ± SEM (N = 10). One-way repeated measures ANOVA; p value was <0.001 for each parameter considered. Tukey’s multiple comparison test.

PAP increased over the 180 min observation time (p<0.001) (Fig 3A) with no significant difference among the reconditioning phase time points (from 45 to 180 min). TPVR significantly decreased during the EVLP procedure (p<0.001) (Fig 3B). After RMs at 40 and 175 min, there was a reduction of Ppeak (Fig 3C, p<0.001), whereas dynamic compliance raised (Fig 3D, p<0.001), with a significant difference at 180 min vs 30, 90, 120, 150, and 45 min vs 30, 120 and 150 (p<0.05).

#### Wet-to-dry weight ratio

The lungs were homogeneously white, compact, with no visible signs of edema or trauma.

Wet-to-dry ratio was similar in EVLP and control group: 5.1 ± 0.2 and 5.0 ± 0.1, respectively (p = 0.903).

## Discussion

The present study describes a successful protocol to perform normothermic ex vivo perfusion using rat lungs. A remarkable outcome was a substantial extension of uninjured ex vivo perfusion to 180 min in the absence of pharmacological interventions. This successful achievement stems from our expertise in both clinical [12–14] and pre-clinical [15, 16] use of the isolated lung. The meticulous procedure description includes videos that show how critical steps were managed and solved.

Clinical EVLP is a major pathway to successful transplantation of marginal lungs. However, this promising technique still requires preclinical investigations able to explore procedural adjustments and/or pharmacological interventions. The model made available in the present research could be a dependable basis for such achievement.

Small animal models of EVLP have a remarkable potential as a factual support to clinical application of this advanced technique. However, translational potential of an effective experimental EVLP requires prolonged perfusion length to reproduce the clinical condition. Indeed, short duration is a weakness of currently used rat models in which uninjured perfusion does not generally exceed 30–120 min (Table 4) [17–25]. This is likely due to rodent lung greater brittleness relative to human or porcine organs and to their tendency to develop atelectasis or edema in a shorter time [8].

**Table 4:** General protocol features and perfusion fluid characteristics of recently reported EVLP rat models.

	General setting			Perfusion solution				
Author, ref	Time, min	Atrium	De-oxygenator	Type	RBC	Antibiotics	Heparin	Anti-inflammatory
**Present model**	180	open	no	in-house	no	yes	yes	no
**Nelson**^**[**^^**22**^^**]**^	60	closed	yes	NR	NR	NR	NR	NR
**Noda**^**[**^^**26**^^**]**^	240	closed	yes	STEEN	NR	yes	NR	yes
**Markou**^**[**^^**29**^^**]**^	140	closed	yes	in-house	yes	NR	yes	NR
**Motoyama**^**[**^^**21**^^**]**^	60	closed	yes	STEEN	NR	NR	yes	NR
**Dacho**^**[**^^**28**^^**]**^	180	open	NR	Krebs-Henseleit	NR	NR	NR	NR
**Pêgo-Fernandes**^**[**^^**30**^^**]**^	60	closed	yes	saline	yes	NR	yes	NR
**Hodyc**^**[**^^**27**^^**]**^	180	closed	no	in-house	NR	NR	NR	yes
**Inokawa**^**[**^^**19**^^**]**^	75	closed	no	In-house	yes/no	NR	NR	NR
**Hirata** ^**[**^^**17**^^**]**^ ^**A**^	120	open	yes^B^	venous blood	yes	NR	yes	NR
**Liu**^**[**^^**23**^^**]**^ ^**A**^	120	open	yes^B^	venous blood	yes	NR	yes	NR

The analysis included rat EVLP protocols recently published.

^A^ Single lung ex vivo perfusion.

^B^ Lung isolated from another rat.

Abbreviations: RBC, red blood cells; NR, not reported.

Attempts to prolong ex vivo perfusion included the use of anti-inflammatory treatments (Table 4). Noda and colleagues [26] showed that addition of methylprednisolone allowed to prolong perfusion up to 4 h, whereas the untreated lung developed edema within 1 h. Similarly, with the use of the anti-inflammatory molecule meclofenamate, the Hodyc’s group [27] was able to prevent edema over 3 h EVLP.

Other studies showed prolonged EVLP without anti-inflammatory therapy but some issues in these experiments deserve consideration (see Tables 5 and 6 for settings of recently reported EVLP rat models). In Dacho’s protocol [28] there was a successful 3 h untreated perfusion, but the procedure involved a very low flow rate that would likely be insufficient to sustain lung metabolism (Table 6). Conversely, Markou and co-workers [29] performed a 140 min EVLP, but unfortunately presence of lung edema was not assessed as this outcome was outside their study aim.

**Table 5:** Comparison of reperfusion phase settings in recently reported rat EVLP protocols.

Author, ref	Temperature, °C	Perfusion		Ventilation					
		Flow, % ^D^	PAP, mmHg	Start, min	VT, % ^D^	Ppeak, cmH_2_O	PEEP, cmH_2_O	RR, bpm	RMs
**Present model**	to 37.5 in 25 min ^B^	from 20 to 100% in 40 min	NR	25	from 70 to 100% in 10 min	NR	3	35	-
**Nelson**^**[**^^**22**^^**]**^	37	to 100% in 15 min	NR	0	to 100% in 15 min	NR	2	NR	sigh
**Noda**^**[**^^**26**^^**]**^	from 20 to 37 in 30 min	from 10 to 100% in 60 min	5–10	20	NR	14–15	5	30	at 25 min
**Markou**^**[**^^**29**^^**]**^	-	-	-	-	-	-	-	-	-
**Motoyama**^**[**^^**21**^^**]**^	37	from 10 to 100% in 10 min	NR	0	NR	-8 ^C^	-4 ^C^	60	NR
**Dacho**^**[**^^**28**^^**]**^	-	-	-	-	-	-	-	-	-
**Pêgo-Fernandes**^**[**^^**30**^^**]**^	NR	from 14–20 to 100% in 10–15 min	<15–20	0	from 25 to 100% in 10 min	NR	NR	60	sigh
**Hodyc**^**[**^^**27**^^**]**^	-	-	-	-	-	-	-	-	-
**Inokawa**^**[**^^**19**^^**]**^	to 37 in 60 min ^B^	NR	<20	60	100%	<30	3	60	NR
**Hirata** ^**[**^^**17**^^**]**^ ^**A**^	37	to 100% in 10 min	NR	0	100%	NR	2	40	NR
**Liu**^**[**^^**23**^^**]**^ ^**A**^	36–38	to 100% in 10 min	NR	0	100%	NR	3	40	at 0 min

Temperature, perfusion and ventilation settings in different research protocols are shown. “Reperfusion” denotes a transient phase in which the lungs are gradually re-warmed, perfused and ventilated until the target values are reached.

^A ^Single-lung ex vivo perfusion.

^B^ Lung temperature.

^C^ Chamber pressure.

^D^ Flow rate and VT are expressed as percent of target value.

Abbreviations: PAP, pulmonary artery pressure; VT, tidal volume; Ppeak, peak inspiratory pressure; PEEP, positive end expiratory pressure; RR, respiratory rate; RMs, recruitment maneuvers; NR, not reported.

**Table 6:** Comparison of reconditioning phase settings in recently reported rat EVLP protocols.

Author, ref	Temperature, °C	Perfusion		Ventilation				
		Flow, ml/min	PAP, mmHg	VT, ml/kg	Ppeak, cmH_2_O	PEEP, cmH_2_O	RR, bpm	RMs
**Present model**	37.5 ^B^	5.7–7.6	NR	7	NR	3	35	at 45 and 175 min
**Nelson**^**[**^^**22**^^**]**^	37	5–10	NR	4	NR	2	NR	sigh
**Noda**^**[**^^**26**^^**]**^	37	16.5 ^D^	5–10	NR	14–15	5	30	-
**Markou**^**[**^^**29**^^**]**^	37	15	10 ^D^	7 ^D^	NR	1–2	NR	sigh
**Motoyama**^**[**^^**21**^^**]**^	37	10	NR	NR	-8 ^C^	-4 ^C^	60	NR
**Dacho**^**[**^^**28**^^**]**^	37	0.08 ^D^	NR	6.2 ^D^	NR	2	60	NR
**Pêgo-Fernandes**^**[**^^**30**^^**]**^	NR	5–7	<15–20	10	NR	NR	60	sigh
**Hodyc**^**[**^^**27**^^**]**^	38	12 ^D^	NR	NR	NR	NR	NR	-
**Inokawa**^**[**^^**19**^^**]**^	37 ^B^	NR	<20	10 ^D^	<30	3	60	NR
**Hirata**^**[**^^**17**^^**]**^ ^**A**^	37	4	NR	5.5 ^D^	NR	2	40	NR
**Liu**^**[**^^**23**^^**]**^ ^**A**^	36–38	3.8 ^D^	NR	4 ^D^	NR	3	40	NR

Temperature, perfusion and ventilation settings in different research protocols are shown. The “reconditioning” phase achieves steady-state perfusion/ventilation.

^A ^Single-lung ex vivo perfusion.

^B^ Lung temperature.

^C^ Chamber pressure.

^D^ Measurement units were aligned.

Abbreviations: PAP, pulmonary artery pressure; VT, tidal volume; Ppeak, peak inspiratory pressure; PEEP, positive end expiratory pressure; RR, respiratory rate; RMs, recruitment maneuvers; NR, not reported.

In the present research, the 180 min-perfusion goal was reached via a stringent procedure optimization without any pharmacological treatment. A key aspect of our protective protocol was the initial phase, during which the steady-state was progressively achieved over a 40-min period. Indeed, although gradual increase in flow rate was adopted by most protocols, only few studies included a slow re-warm of the lung and a delayed beginning of ventilation (Table 5) [22, 26, 30]. The present model performed a stepwise enhancement of three crucial parameters: temperature, flow rate, and tidal volume. Expressly, temperature was gradually augmented because lungs were in hypothermic conditions after ice-cold flushing with Perfadex®. We recommend assessing of the actual temperature of perfusion fluid, possibly in close proximity to the graft. Flow rate was also increased stepwise to reduce hemodynamic stress. Of note, in order to avoid hydrostatic edema, we elected to use a moderately low flow rate coupled with an open atrium strategy [13], though this approach is typically associated with high flow rates in the clinical setting [1]. Ventilation was started only when lung temperature reached the physiological range. We used a protective ventilation strategy with a respiratory rate corresponding to half the physiological value. Tidal volume was initially low and increased progressively. The use of a gas mixture of room air supplemented with 5% CO_2_ enabled to overcome the lack of a membrane (de)oxygenator. Recruitment maneuvers were carried out twice during the procedure to expand de-recruited areas.

This protocol was clearly adequate to sustain lung function, as indicated by gas analysis and functional outcome assessments. Indeed, perfusate electrolyte composition as well as glucose consumption and lactate production indicated active metabolism. In addition, airway and pulmonary pressure remained in a physiological range during ex vivo perfusion. Finally, at the end of the procedure, W/D ratio of perfused lungs was similar to that of controls, indicating absence of edema.

Other important aspects had to be addressed in order to perform reproducible and successful ex vivo perfusion.

The use of a surgical microscope allowed a better visualization of the pulmonary artery and detection of gas embolism that represents the leading cause of non-homogeneous perfusion patterns [22]. We also recommend the use of a pulmonary artery cannula equipped with a bubble trap.

Though in EVLP rat models lungs are usually suspended from the tracheal and pulmonary cannulae [19, 21, 22, 26, 27, 29, 30], we elected to put the graft horizontally, similar to the clinical setting [1]. However, because rat lungs are fragile, we used an ad hoc modeled elastic surface to avoid excessive pressure to soft tissues.

The perfusion fluid used in this model has the same principal components of STEEN solution™ that is generally used in the clinical setting [1]. The fluid consisted of an extracellular-type solution supplemented with human serum albumin to reduce edema development, heparin to avoid clot formation, dextran to prevent leukocyte adhesion [31], and glucose as energy source. Moreover, unlike the majority of EVLP rat protocols (Table 4), we added antibiotics to the perfusion solution and checked for bacterial contamination at the end of each procedure.

In clinical EVLP, the perfusate is replaced periodically to preserve lung function [13, 32]. However, we elected not to perform solution substitutions in order to evaluate concentration of inflammatory mediators, cells, electrolytes, and metabolites over time. Interestingly, electrolyte and metabolite content was similar in samples collected from auricle and reservoir. On the other hand, reservoir pO_2_ was lower relative to auricle pO_2_ because of gas exchange between perfusion fluid and ambient air. Therefore, auricle withdrawal—that is unavoidably associated with temperature and humidity perturbations caused by opening of lung chamber–is essential for pO_2_ assessment but could be omitted to evaluate electrolyte or metabolite concentration.

To facilitate reproduction of our model by other laboratories, instructional videos are provided. Indeed, direct observation of the crucial steps hastens improvement of learning curves [33], in compliance with the 3Rs principles (Refinement, Reduction and Replacement) [9].

In conclusion, this paper provides a detailed description of a small animal model of EVLP marked by prolonged ex vivo perfusion, optimal lung function, and absence of injury. This goal was achieved through a stepwise modification of the protocols in use. The detailed description of the approach to individual problems can help researchers to reproduce the procedure.

A reliable preclinical model could be very helpful to improve ex vivo perfusion techniques in both standard and marginal lungs and to investigate novel therapeutic intervention before transplantation.

## Supporting Information

S1 Fig Bland-Altman plots. Agreement between measurements performed using auricle and reservoir perfusate samples. Panel A: linear regression analysis. Panel B: Bland–Altman analysis. Y-axis represents the difference between auricle and reservoir evaluations, while X-axis represents the mean of the two measurements. Horizontal lines represent the mean difference (solid lines) and the limits of agreement calculated as mean difference ± 2 times the standard deviation (dashed lines).
(PDF)

S1 Video In situ lung function evaluation. Assessment of total lung capacity (TLC) and basal elastance after performing a recruitment maneuver.
(MP4)

S2 Video In situ lung perfusion. Incannulation of pulmonary artery and resection of auricles and heart apex.
(MP4)

S3 Video In situ lung perfusion. Flushing of lungs with Perfadex®.
(MP4)

S4 Video Heart-lung block harvest. Surgery for organ procurement.
(MP4)

S5 Video Isolated lung perfusion system. Overview of ex vivo lung perfusion setting.
(MP4)

S6 Video Isolated lung perfusion protocol. Ventilation and recruitment maneuver during ex vivo perfusion.
(MP4)
