# Supplementary material for: Ex Vivo Lung Perfusion in the Rat: Detailed Procedure and Videos
Source: PLoS One. 2016 Dec 9;11(12):e0167898. doi: 10.1371/journal.pone.0167898 (PMC5148015; doi:10.1371/journal.pone.0167898)

pH

A

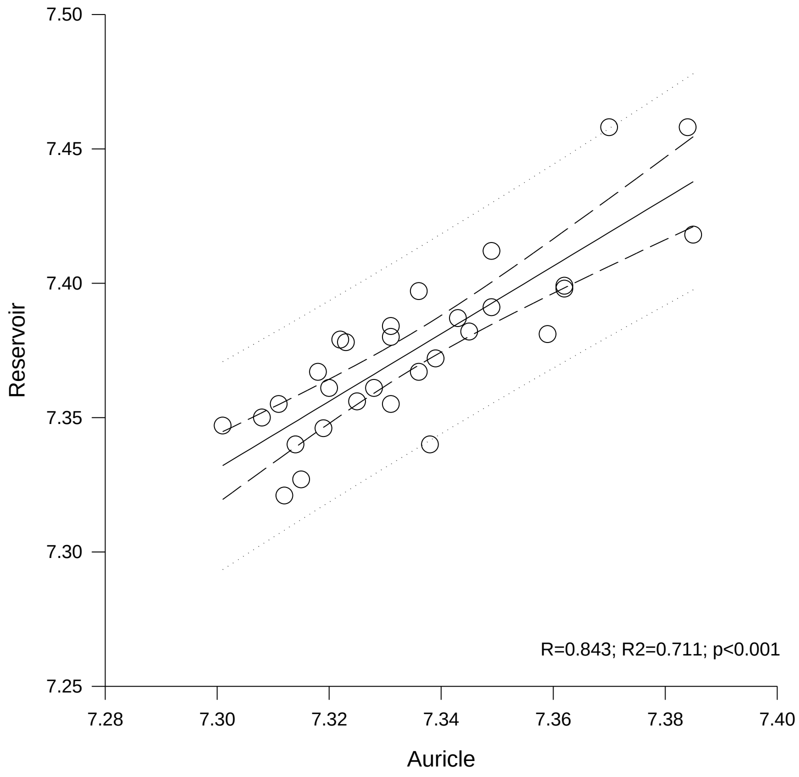

7.50
7.45
7.40
7.35
7.30
7.25
Reservoir
7.28
7.30
7.32
7.34
7.36
7.38
7.40
Auricle
R=0.843; R2=0.711; p<0.001

B

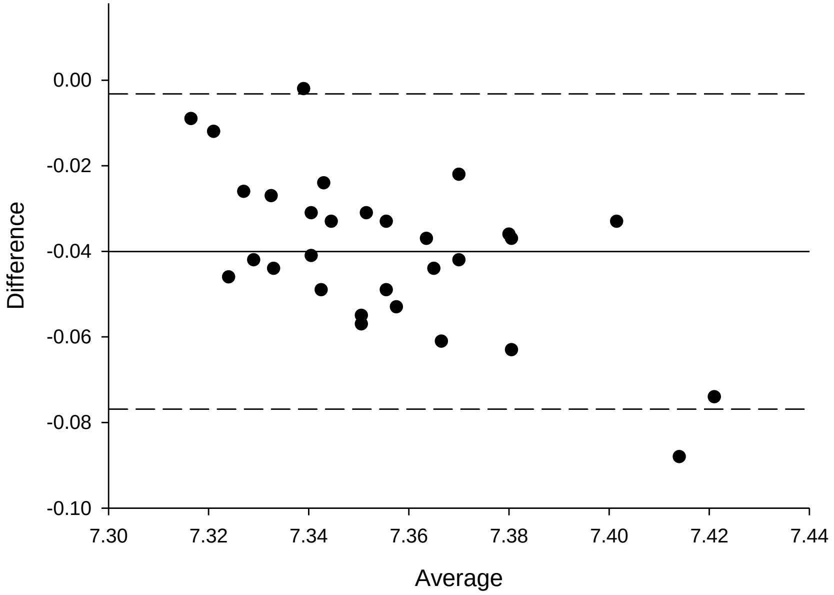

0.00
-0.02
-0.04
-0.06
-0.08
-0.10
Difference
7.30
7.32
7.34
7.36
7.38
7.40
7.42
7.44
Average

pO_2, mmHg

A

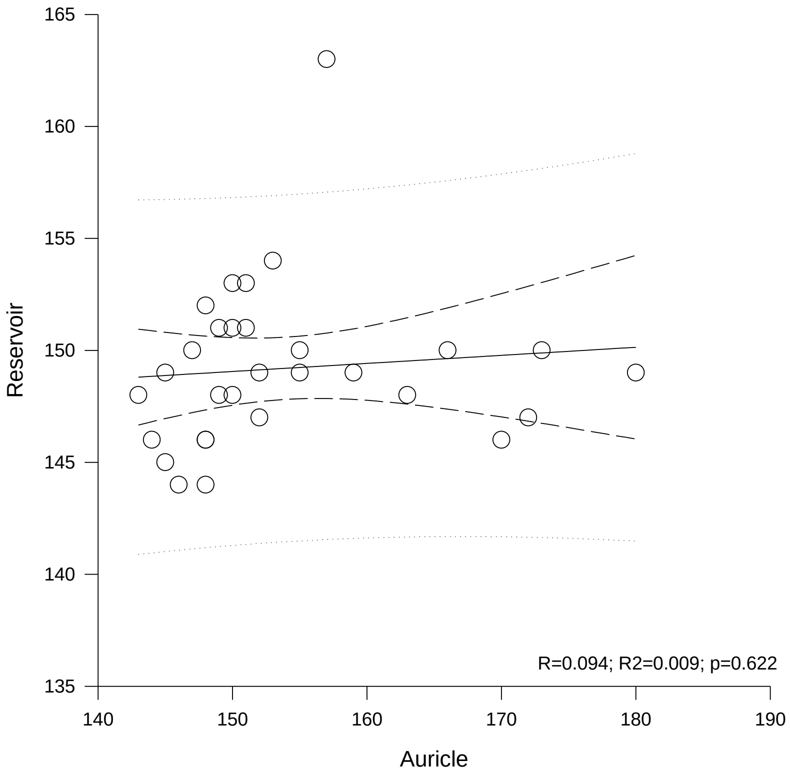

165
160
155
150
145
140
135
Reservoir
140
150
160
170
180
190
Auricle
R=0.094; R2=0.009; p=0.622

B

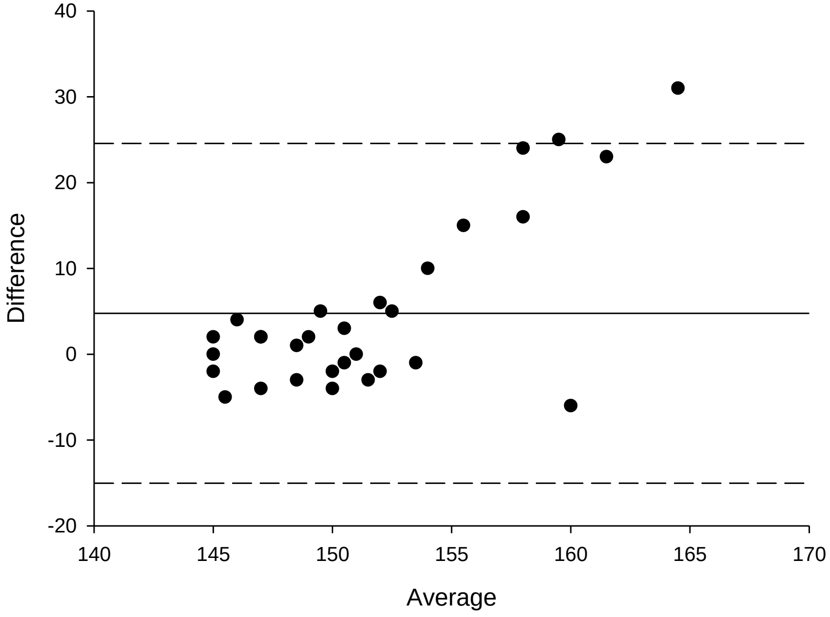

40
30
20
10
0
-10
-20
Difference
140
145
150
155
160
165
170
Average

pCO_2, mmHg

A

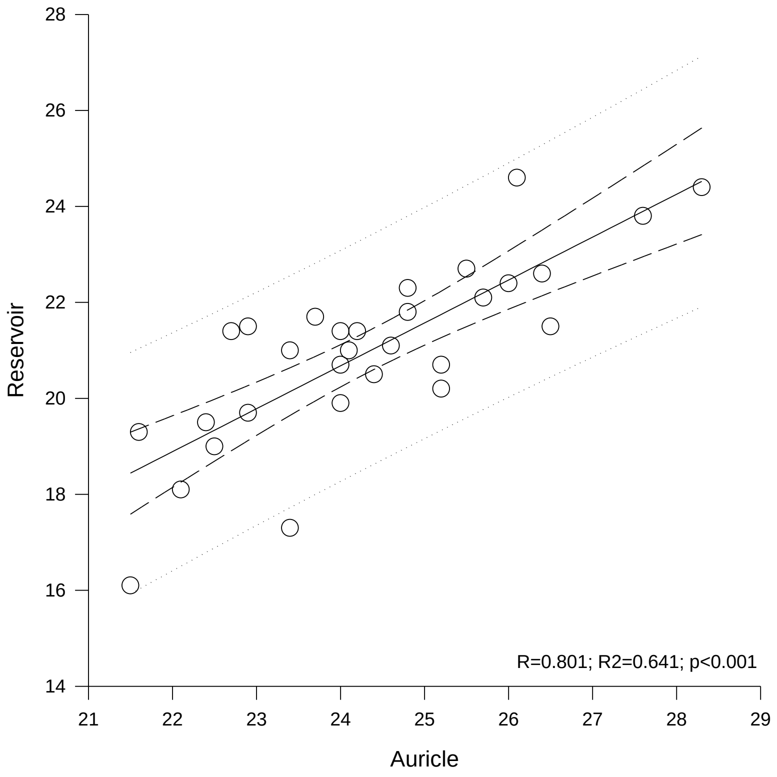

28
26
24
22
20
18
16
14
21
22
23
24
25
26
27
28
29
Reservoir
Auricle
R=0.801; R2=0.641; p<0.001

B

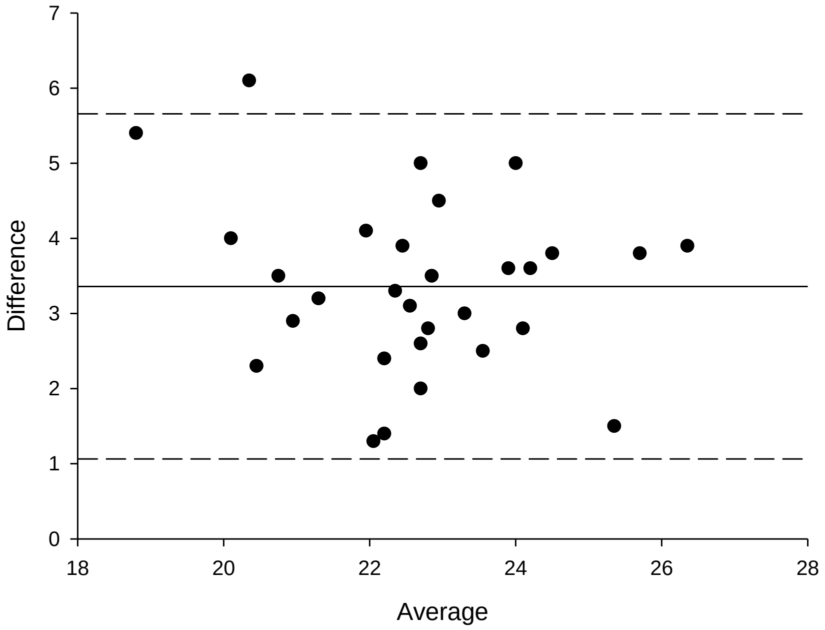

7
6
5
4
3
2
1
0
18
20
22
24
26
28
Difference
Average

K^+, mmol/L

A

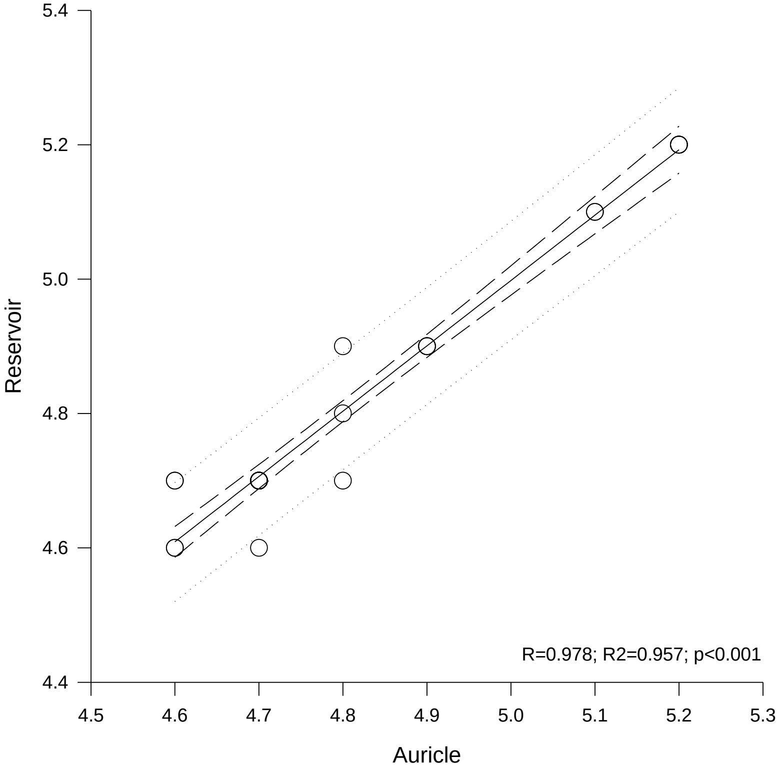
Reservoir
Auricle
R=0.978; R2=0.957; p<0.001
5.4
5.2
5.0
4.8
4.6
4.4
4.5
4.6
4.7
4.8
4.9
5.0
5.1
5.2
5.3

B

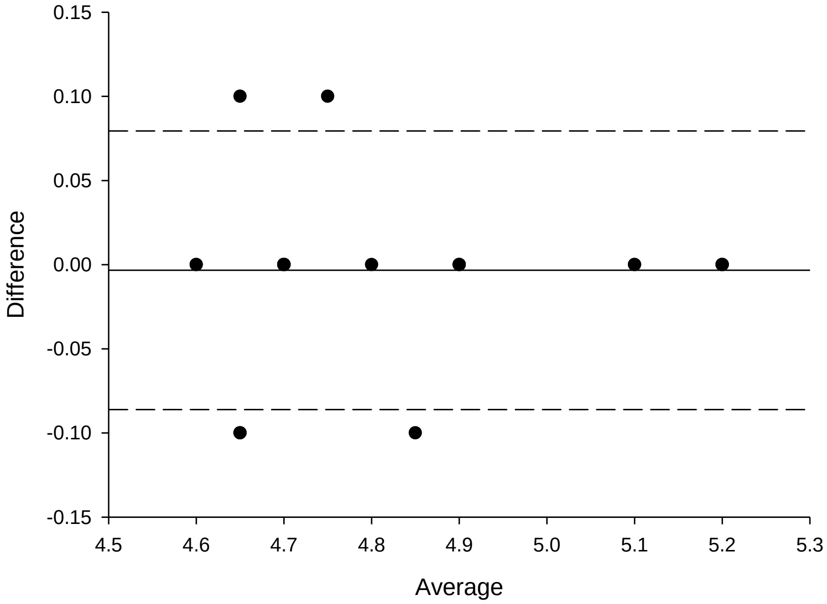
Difference
Average
0.15
0.10
0.05
0.00
-0.05
-0.10
-0.15
4.5
4.6
4.7
4.8
4.9
5.0
5.1
5.2
5.3

Na^+, mmol/L

A

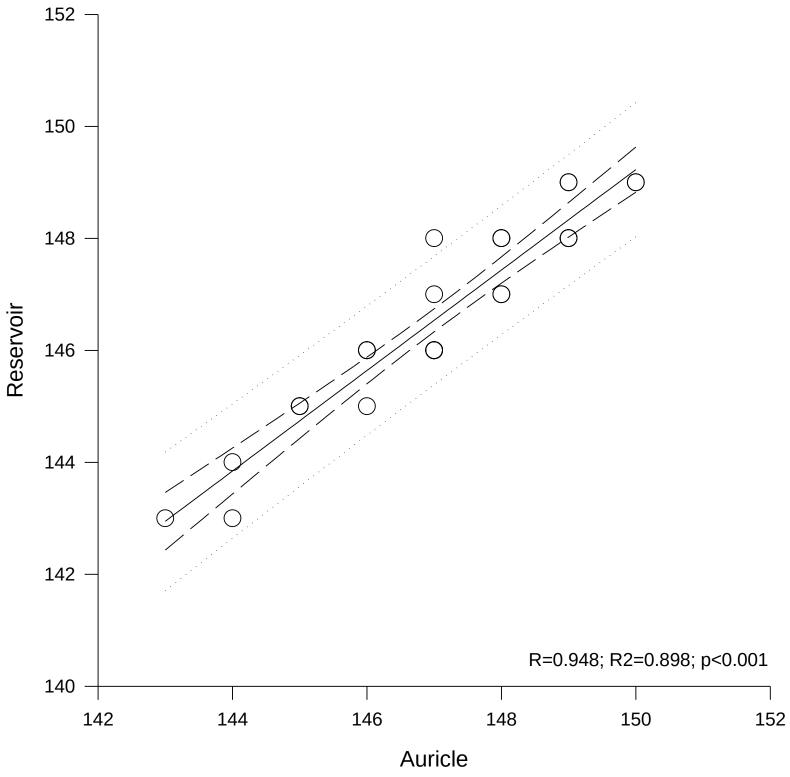
152
150
148
146
144
142
140
Reservoir
142
144
146
148
150
152
Auricle
R=0.948; R2=0.898; p<0.001

B

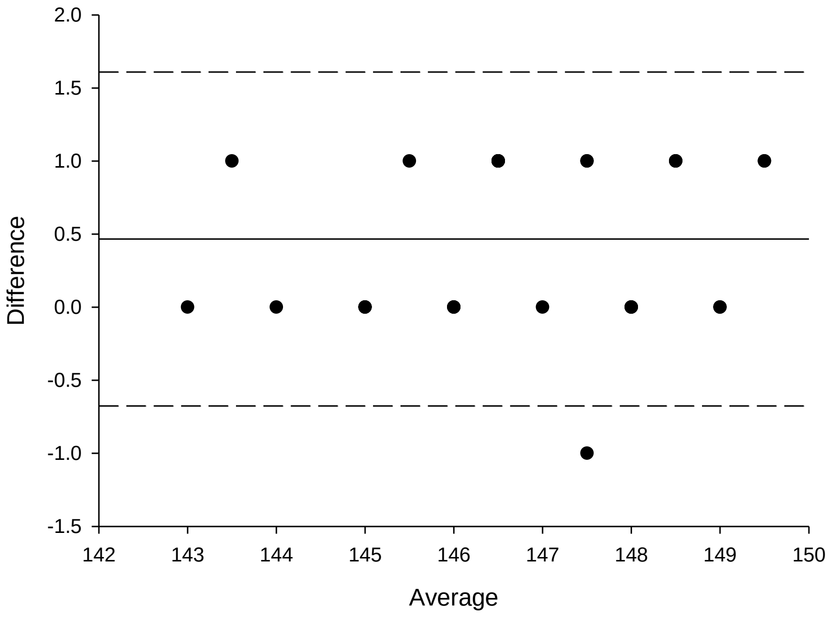
2.0
1.5
1.0
0.5
0.0
-0.5
-1.0
-1.5
Difference
142
143
144
145
146
147
148
149
150
Average

Ca^{2+}, mmol/L

A

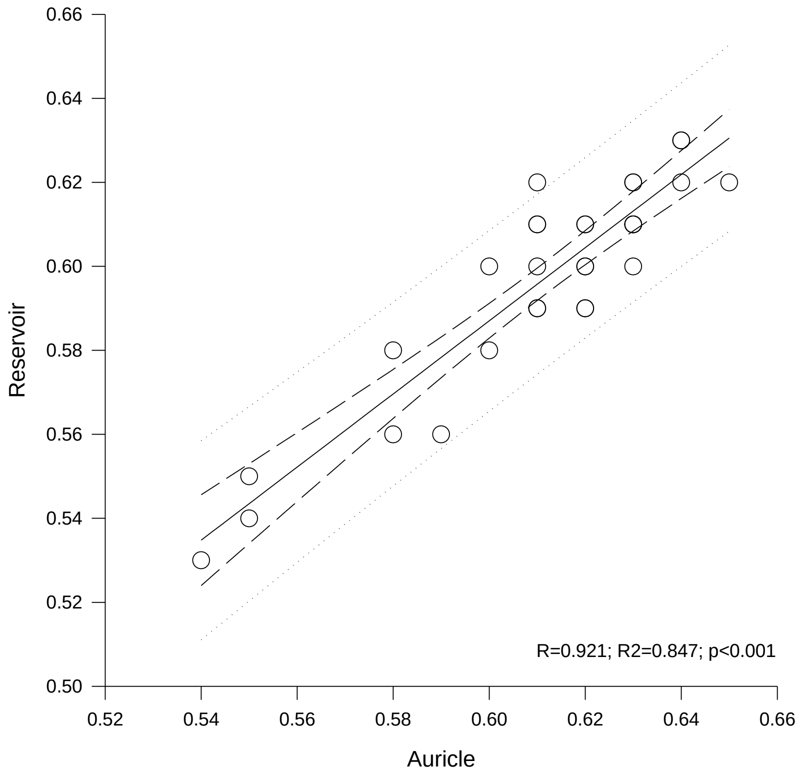

Reservoir
Auricle
R=0.921; R2=0.847; p<0.001
0.50
0.52
0.54
0.56
0.58
0.60
0.62
0.64
0.66

B

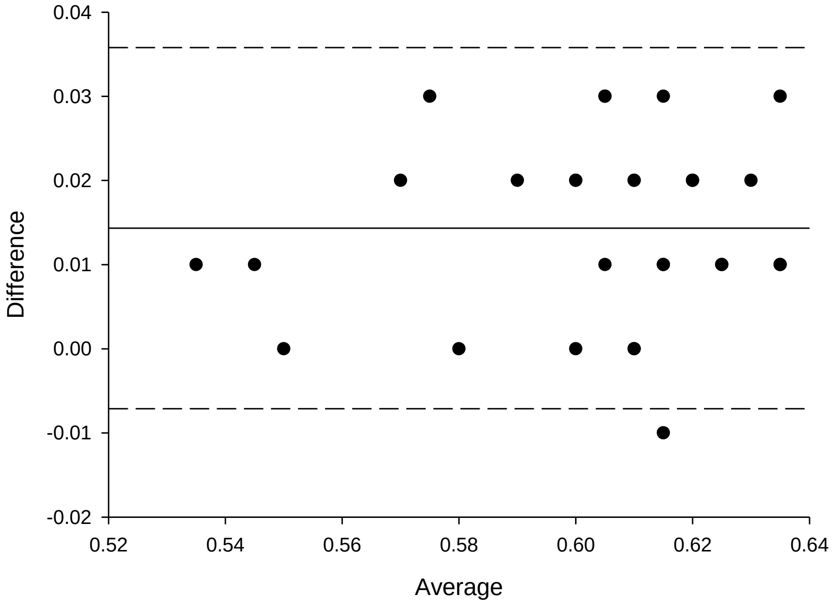

Difference
Average
-0.02
-0.01
0.00
0.01
0.02
0.03
0.04
0.52
0.54
0.56
0.58
0.60
0.62
0.64

Cl^-, mmol/L

A

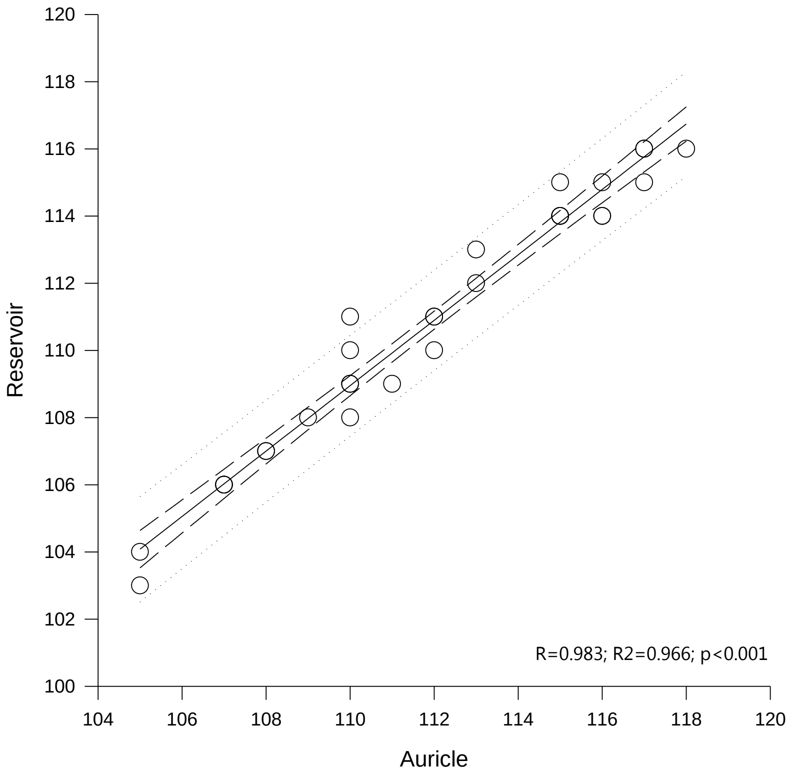

Reservoir
Auricle
R=0.983; R2=0.966; p<0.001
120
118
116
114
112
110
108
106
104
102
100
104
106
108
110
112
114
116
118
120

B

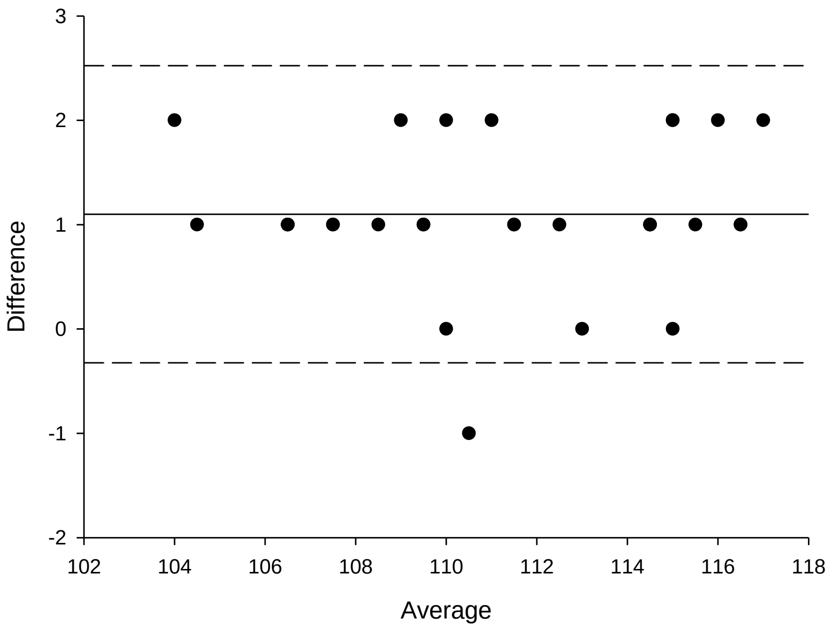

Difference
Average
3
2
1
0
-1
-2
102
104
106
108
110
112
114
116
118

Glucose, mg/dl

A

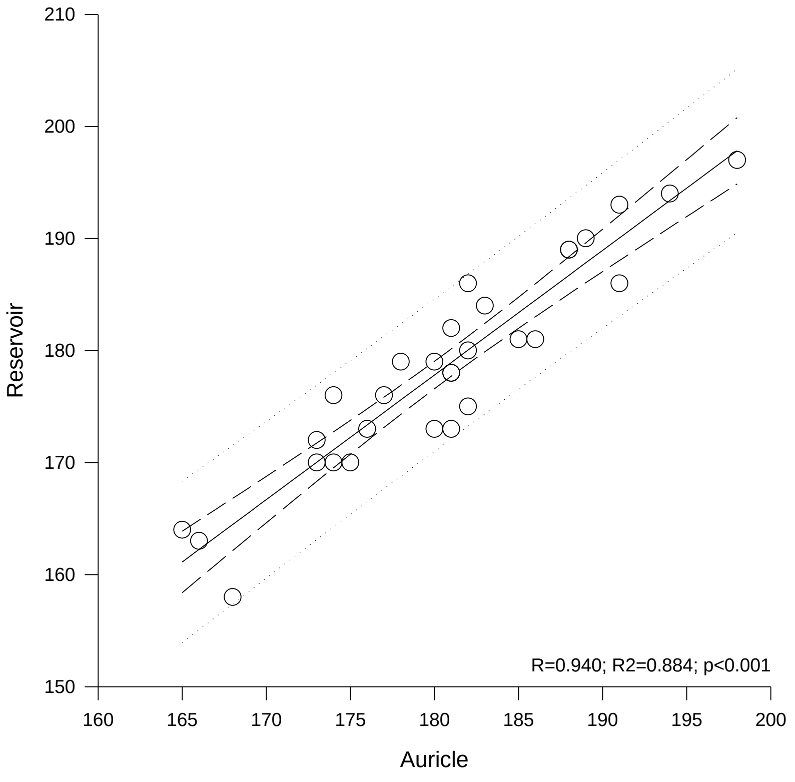

B

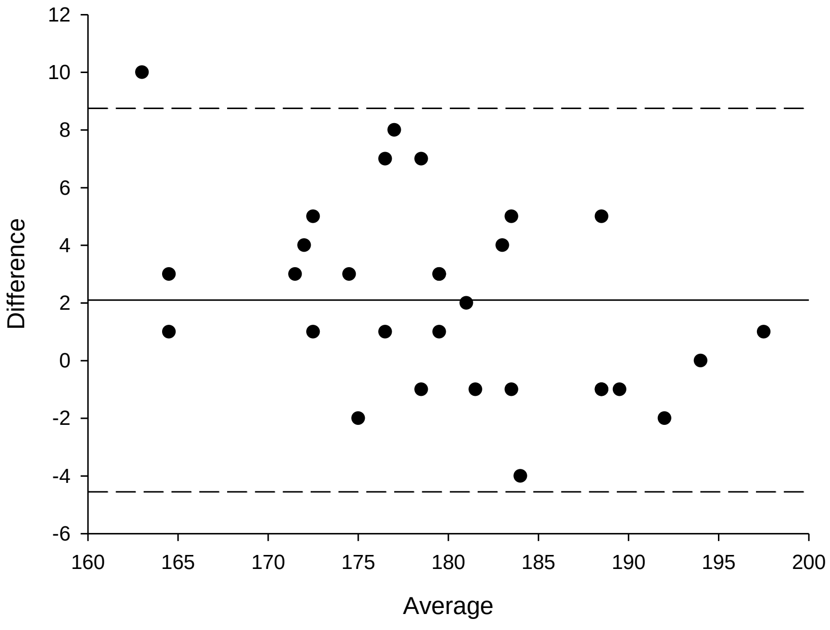

Lactate, mmol/L

A

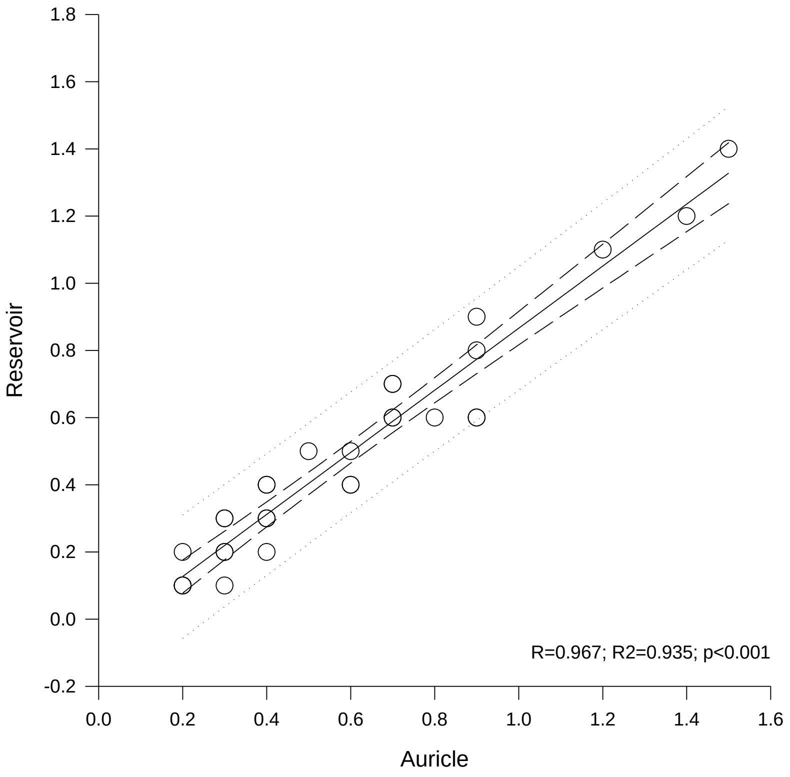

B

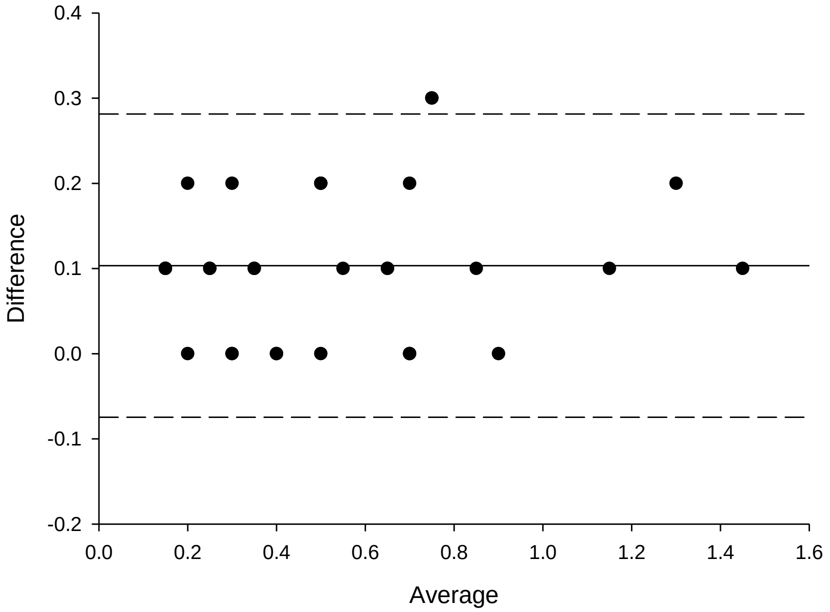

Supplement: S1 Fig — Agreement between measurements performed using auricle and reservoir perfusate samples. Panel A: linear regression analysis. Panel B: Bland–Altman analysis. Y-axis represents the difference between auricle and reservoir evaluations, while X-axis represents the mean of the two measurements. Horizontal lines represent the mean difference (solid lines) and the limits of agreement calculated as mean difference ± 2 times the standard deviation (dashed lines). (PDF) [file pone.0167898.s001.pdf]
